# How Do We Clean Up the Scientific Record?

**DOI:** 10.1093/function/zqad055

**Published:** 2023-10-12

**Authors:** Alexei Verkhratsky, Ole H Petersen

**Affiliations:** Faculty of Biology, Medicine and Health, University of Manchester, Manchester M13 9PT, UK; School of Biosciences, Sir Martin Evans Building, Cardiff University, Cardiff CF10 3AX, UK

**Keywords:** scientific errors, correction of errors, fraud, irreproducibility, wrong methodology

The famous phrase “Errare humanum est, sed in errare perseverare diabolicum” (“To err is human, but to persist in error is diabolical”), which is attributed to the Roman philosopher and orator Lucius Annaeus Seneca (∼4 bce to 65 ce), is as relevant today as it always was. It is particularly important for science. We all make mistakes, but we should be careful not to persist in error and, most importantly, do everything we can to correct our errors and do so as quickly as possible. Otherwise, our scientific record will be unreliable and therefore not a secure basis for further work and rational decision making. In the worst cases, real harm is done to the care of patients.

The problem is often dealt with under the heading of irreproducibility, but it is really a question of getting it right. There are many examples of incorrect findings that were perfectly reproduced by repeating the mistakes or wrong assumptions others had made. In extreme cases, fraud or glaring errors, published papers are usually retracted and the scientific record is therefore cleaned up. However, there are unfortunately many more cases in which seriously flawed articles remain uncorrected. Although new papers may appear that correct erroneous articles previously published, the wrong papers usually remain part of the literature and may continue to cause confusion.

In this issue of *FUNCTION*, Anant Parekh and colleagues from NIH/NIEHS, North Carolina, publish a salient paper^1^ that corrects a serious error in a previously published article^2^ that has important consequences for the treatment of hypertension, the leading cause of death globally, accounting for >10 million deaths annually.^3^ The issue concerns the mechanism of action of amlodipine, a dihydropyridine blocker of voltage-gated L-type Ca^2+^ channels, a first-line choice for the treatment of hypertension.^3^ It has for a long time been generally accepted that amlodipine specifically inhibits opening of L-type Ca^2+^ channels, thereby reducing the cytosolic Ca^2+^ concentration in vascular smooth muscles, which, in turn, will relax and widen the blood vessels. However, in a paper published in July 2020, Johnson et al. appeared to have shown that amlodipine triggers store-operated Ca^2+^ entry, thereby increasing the cytosolic Ca^2+^ concentration in vascular smooth muscle cells.^2^ They concluded that: “These results provide unique mechanistic insights into how widely used drugs activate a Ca^2+^ signaling pathway and suggest that the use of L-type Ca^2+^ channel blockers in patients with chronic hypertension, where levels of STIM proteins and vascular remodeling are already enhanced, should be avoided.”^2^ Fortunately, for the many patients who are treated with amlodipine, it turns out that the results reported by Johnson et al.^2^ are wrong. Johnson et al. used Fura-2 to measure the cytosolic Ca^2+^ concentration and Anant Parekh and his collaborators now show that amlodipine has marked intrinsic fluorescence, over an excitation spectrum that is identical to that of Fura-2.^1^ Using longer wavelength Ca^2+^ indicators, they show that amlodipine, in concentrations that correspond to therapeutic levels in patients, does not activate store-operated Ca^2+^ entry.^1^ The finding of Johnson et al.^2^ is therefore an artifact, based on a failure to check a critical chemical property of amlodipine, namely its intrinsic fluorescence, overlapping with that of Fura-2.^1,3^ As Rajagopal and Rosenberg point out in their commentary published in this issue,^3^ the clinical utility and safety of amlodipine is now no longer in question, thanks to the rigorous work reported by Parekh and colleagues. This case highlights, as previously discussed,^4^ that the most critical issues with reports of experimental work, contrary to the general belief, are not poor statistics or lack of adherence to guidelines, but simply flawed methodology and absence of appropriate controls.

Observations, trials, errors, new trials, (mis)interpretations, revisits, and reconsiderations have been the path of science since the early days of Greek philosophy. Aristotle, who was the first to produce a comprehensive description of the brain (he distinguished between cerebrum and cerebellum, discovered the dura and pia mater, meninges, and brain vessels, and most likely was the first to see brain ventricles), regarded the brain as a mere cooler for the psychic pneuma originating in the heart and distributed through the circulation to govern the body. Galen, the father of experimental neurosciences, placed the pneuma in the ventricles, believing that the rest of the brain was the sole producer of this pneuma from the air. Franz Josef Gall, who contemplated the localization of brain function, created phrenology, which captured the world, but it was subsequently ridiculed and has now been reborn at a completely new level with advances of brain imaging. The list of misconceptions and scientific mistakes is endless and is part of the progression of knowledge: Past errors are the seeds of future discoveries. However, this seemingly inexorable spiral ascent requires academic transparency, critical appraisal, acceptance of errors, and their swift rectification.

Drastic changes in reporting scientific data have seen an exponential increase in publications over the last decades representing a major threat to the classical model tested over millennia. Not only have millions of published reports increased the noise level through repeating trivial observations or conducting useless experiments, but the avalanche of papers has made their post-publication critical appraisal, testing, reproduction, and, most importantly, rectification almost impossible. This applies to both fraud and misconduct as well as honest errors that are inevitable in scientific research. As argued by John Ioannidis,^5^ “most published research findings are false,” the reasons varying from objective (preparations, methods, effect sizes) or lack of knowledge (lack of definitions, ignorance of the literature) to personal (bias linked to funding, exposure, and career progression). The rise of predatory publishers with little or no quality control facilitates multiplication of squander. The situation is further exacerbated by a substantial increase in scientific fraud ranging from data manipulation to their outright invention; what we can identify is most likely only the tip of the iceberg. Similarly, plagiarism (which fortunately is easier to spot through the use of modern software) plagues academic writings from student essays to papers in reputable journals. The reason is simple—the resources for testing and assessing scientific work are woefully insufficient, thus triggering wild competition that erodes academic morale already shaken by poor training of an exponentially rising number of students.^6^

Academia faces a crisis; lack of reproducibility and lack of trust make further progress questionable. What are the tools and remedies? Surprisingly, these are rather few. As already mentioned, fraud can at least be identified, and the fraudulent papers withdrawn; plagiarism can be dealt in the same way, but how to rectify straightforward errors, which (as illustrated by the case discussed in this editorial) carry dangerous implications? The only way is post-publication testing of the main findings and, in the case of error identification, making the academic community informed through publication, which is of course exactly what is happening in the case discussed here. However, this pathway is severely handicapped, because the modern environment does not favor publication of negative results. There is little place for open and transparent critical discussion of published data. The ideal solution is of course that an author accepts errors made and publishes a correcting paper. The most famous paper of Sydney Ringer, demonstrating the fundamental role of Ca^2+^ in the contraction of the heart,^7^ is an illustrious example, as it repudiated his own results published a year earlier.^8^ Whether academic decency will prevail in our brave new world, or we succumb to senseless overproduction of irrelevant or erroneous papers, is the main challenge that will define future academic progress.

We must find practical ways to deal with this problem, and discussion about how to do this has started.^9^ We now need a serious debate about the merits and perils of various measures. As mentioned in an earlier editorial in another journal,^4^ certain “cures” could be worse than the “disease.” No doubt we, and many others, will return to this theme on many occasions in the future.

## Data Availability

There are no data in this editorial.
